# Enhancing the Strengthening Effect of Graphene-Nanoplates in Al Matrix Composites by Heterogeneous Matrix Design

**DOI:** 10.3390/nano12111833

**Published:** 2022-05-27

**Authors:** Puzhen Shao, Kai Sun, Ping Zhu, Kai Liu, Qiang Zhang, Wenshu Yang, Zhijun Wang, Ming Sun, Dingyue Zhang, Sergey Kidalov, Haiying Xiao, Gaohui Wu

**Affiliations:** 1 Department of Material Science and Engineering, Harbin Institute of Technology, Harbin 150001, China; shaopuzhen@163.com (P.S.); 15776720450@163.com (K.S.); 18846450834@163.com (P.Z.); kailiou2035@gmail.com (K.L.); hitzhijun@gmail.com (Z.W.); s1257973295@163.com (M.S.); 17b909059@stu.hit.edu.cn (D.Z.); wugh@hit.edu.cn (G.W.); 2 Laboratory “Physics for Cluster Structures”, Ioffe Institute, 194021 Saint-Petersburg, Russia; kidalov@mail.ioffe.ru

**Keywords:** metal-matrix composites (MMCs), mechanical properties, microstructures, finite element analysis (FEA)

## Abstract

In the present work, the properties of graphene-nanoplates/aluminum (GNPs/Al) composites with a heterogeneous matrix design were investigated. The advantage of the heterogeneous matrix was investigated by the finite element method. Then, 0.6 wt.% (GNPs/6061Al)/2024Al (heterogeneous matrix) and 0.6 wt.% GNPs/6061Al composites were prepared by ball milling, pressure infiltration technology, and hot extrusion. The aggregation of GNPs was eliminated and the interlayer slide of GNPs was observed. Mechanical property test results show that the mechanical properties of the heterogeneous matrix composite are better than that of a homogeneous matrix composite, including strength, elastic modulus, and plasticity. It is assumed that the heterogeneous matrix design enhances the non-uniform stress field during the deformation treatment. This improves the dispersion of GNPs, grain refinement, and produces the few-layer graphene (FLG), thus enhancing the strengthening effect of GNPs. Meanwhile, heterogeneous matrix design is thought to introduce more hardening mechanisms to increase the plasticity of materials and improve the intrinsic trade-off of strength and toughness.

## 1. Introduction

Recently, GNPs/aluminum (Gr/Al) composites have been thoroughly studied, and have made great progress. The addition of GNPs can significantly improve the properties of the matrix, which has attracted the wide attention of researchers [1,2,3,4,5]. Wang et al. [6] prepared 0.3 wt.% graphene nanosheet/Al (GNS/Al) composites by chemical adsorption and hot-pressing sintering, and its tensile strength was 62% higher than that of the matrix; Liu et al. [7] prepared Ni precursor, by the template method, for in situ growth of GNPs, and synthesized Ni-NPs@GNP using chemical vapor deposition (CVD), then fabricated 0.7 vol.% Ni-NPs@GNP/6061Al by means of powder metallurgy. The yield strength of the composite is 75% higher than that of the pure 6061Al matrix.

The results show that the strengthening behavior of GNPs is related to their dispersion and specific surface area. Graphene has a large specific surface area (the specific surface area of single-layer graphene is about 2630 m^2^/g), which is very easy to agglomerate [8]. In this case, GNPs are not beneficial for the improvement of composite’s properties, while they will become a source of cracks, leading to crack propagation of the composite under load. Rashad et al. [9] studied the microstructure and mechanical properties of GNPs/Al composites with different contents. It was found that when the content of GNPs was high, it would stack into graphite particles due to the effect of the π–π bond between layers, which led to the decrease in material plasticity. Shin et al. [10] compared and analyzed the strengthening behavior of GNPs and carbon nanotubes in Al matrix composites, and found that they can be described by the modified shear lag model (Equation (1)),

(1) $$\sigma_{c}=V_{r}(\frac{S}{A})(\frac{\tau_{m}}{2})+\sigma_{m}V_{m}$$

where *σ_c_* and *σ_m_* are the yield strength of the composite and matrix, *V_r_* and *V_m_* are the volume fraction of carbon nanomaterials and matrix, respectively, *S* is the contact interface area, *A* is the cross-sectional area, and *τ_m_* is the interfacial shear strength of the matrix. It can be seen that the larger diameter and thinner thickness of GNPs will make a more obvious contribution to the strengthening effect under the condition of uniform dispersion and the same content. 

In recent years, the preparation process of GNPs/Al composites mainly includes pre-dispersion treatment, consolidation, and deformation treatment, including hot extrusion, hot rolling, and severe plastic deformation, etc. [11,12,13]. Among them, pre-dispersion treatment and deformation treatment can play a role in dispersing and producing few-layer graphene (FLG). More in-depth research on GNPs pre-dispersion has been carried out at home and abroad, including ball milling [14,15,16,17], molecular level mixing method [18], CVD [19,20], etc. In the current research, the pre-dispersion treatment is mainly realized by mechanical ball milling. Researchers have made significant research on the process parameters such as milling speed [21], time [22], and ball milling program [23]. 

As a conventional treatment process of composite materials, deformation treatment has also been widely used in the preparation of GNPs/Al composites [24,25,26]; however, in order to improve the mechanical properties of the composites, further research is needed. At present, research on GNPs/Al composites is mainly focused on the homogeneous aluminum matrix [27]. In the process of deformation treatment, the stress field of the matrix forms a shear effect on GNPs under the external load so as to eliminate the GNPs agglomeration and realize the peeling and delamination of graphene nanoplates; therefore, the formation of asymmetric strain behavior on two sides of the GNPs is the key to promote the dispersion and peeling of GNPs. In addition, the design of heterogeneous materials is of great significance for the improvement of material properties, including plasticity and elastic modulus. Salama et al. [28] prepared double matrix carbon nanotube/Al (CNTs/Al) composites. The research results show that compared with the uniform configuration, the non-uniform structure design can improve the plasticity and elastic modulus of the composite; Wu et al. [29] used the magnetron sputtering method to uniformly disperse 6 nm nanocrystalline MgCu_2_ into the amorphous Mg_69_Cu_11_Y_20_ shell and obtained 3.3 GPa ultra-high strength, which is close to the theory. Huang et al. [30] proposed the idea of “micro inhomogeneous”, and prepared TiBw/Ti composites with a quasi-continuous network distribution of reinforcements via the in situ method, which greatly improved the strength and plasticity of traditional titanium matrix composites and caused researchers to think about the non-uniform design of composites and other materials [31]. 

In the present work, we focus on eliminating the aggregation of GNPs via the deformation treatment. It should be emphasized that the deformation treatment method used in our research is hot extrusion, which can produce flow deformation in the material, including compression, shear, and their mixing effects. These effects can separate the agglomerated GNPs or reduce the number of layers of GNPs. The stress field of the heterogeneous matrix under the condition of thermal deformation was researched using the finite element method (FEM). In addition, the effect of property differences of heterogeneous matrix components on the strain field of two-dimensional reinforcement was discussed. We chose the 2024Al and 6061Al, which were widely used in the preparation of AMC, as the matrix to research the heterogeneous matrix composite. Based on the unique advantages of pressure infiltration technology, a heterogeneous composite (GNPs/6061Al)/2024Al was prepared. The heterogeneous matrix design is considered to further enlarge the asymmetric strain on both sides of GNPs, so as to promote dispersion, peeling, and delamination of GNPs. In addition, we discussed the potential benefits of heterogeneous design by comparing the properties differences between GNPs/6061Al and the homogeneity composite.

## 2. Materials and Methods

### 2.1. Finite Element Method (FEM) Simulation 

The finite element method simulation was performed on Abaqus to study the deformation characterization of GNPs/Al alloy composite. Considering the stress state of the aluminum rod during extrusion, a 2-D model with plane stress conditions was applied. The geometry structure for the composite was simplified into a three-layer model, in which a sheet of GNPs was embedded at the interface between two aluminum blocks. The model is 30 × 30 µm^2^. The size of the GNPs sheet is 10 × 0.5 µm^2^.

The GNPs sheet was considered as an isotropic elastomer, with Young’s modulus of 1000 GPa and Poisson’s ratio of 0.1. The aluminum is considered an elastoplastic, with Young’s modulus of 70 GPa and Poisson’s ratio of 0.3. The same multi-linear kinematic hardening model is applied for aluminum alloys with yield stress varying from 1 to 300 MPa. Since the simulation aimed to show the non-uniform strain distribution at both sides of the GNPs sheet induced by the difference in the matrices’ strength during deformation, the work hardening curve of aluminum alloys is artificially designed to facilitate computational convergence, as is shown in Table 1. Considering the excellent deformability of aluminum alloys during high-temperature extrusion, the assumption is acceptable. The extrusion die was modeled with discrete rigid wire. The extrusion ratio during the simulation was 3:2.

The model was meshed with 4-node bilinear quadrilateral elements with reduced integration (CPS4R). The bonding between GNPs and aluminum was set to be perfect. Frictionless contact pairs were defined between the workpiece and the extrusion die. The extrusion was performed by applying a constant velocity of 10 µm/s to the top plane of the workpiece. Explicit dynamic simulations were performed on a series of models with various matrix strengths. Since the extrusion can be taken as a quasi-static process, mass scaling was applied to accelerate the computation.

**Figure 1:**
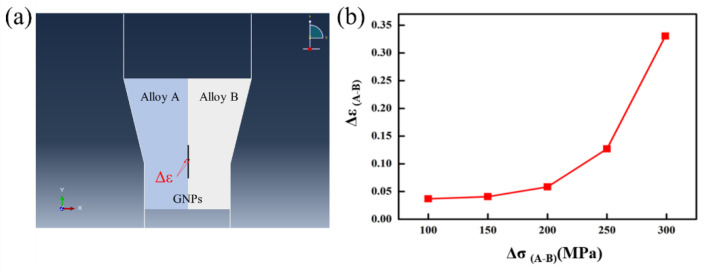
FEM simulation of the effect of heterogeneous matrix on the deformation behavior of GNPs: (**a**) model diagram; (**b**) strength difference–strain curve.

In the postprocessor, the shear strain at two sides of the GNPs sheet is extracted. The difference in the strain is plotted with respect to the difference in the matrices’ yield strength, as is shown in Figure 1a. Considering a much higher extrusion ratio was applied in experiments, a more significant strain mismatch could be expected. The corrected mechanical properties of GNPs and Al alloy in the models are shown in Table 2. 

### 2.2. Materials and Preparations 

**Figure 2:**
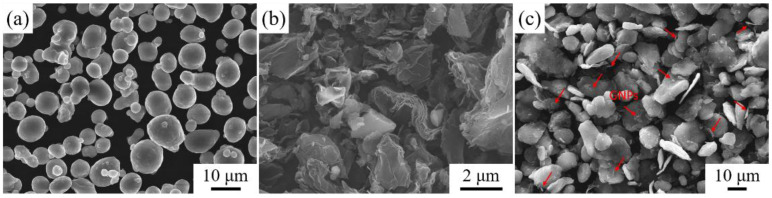
Microstructure of the raw materials used in this work: (**a**) SEM image of the 6061Al powder; (**b**) SEM image of GNPs; (**c**) mixed powder after ball milling. Some GNPs have been marked.

The 6061Al powders had an average diameter of 7.5 μm (Figure 2a) and the 2024Al alloy pure Al was supplied by Northeast Light Alloy Corp. Ltd. (Harbin, China), while the GNPs (Figure 2b) were supplied by the Sixth Element Materials Technology Co. Ltd. (Changzhou, China). Chemical compositions of the 6061Al powder and 2024Al have been listed in Table 3. 

**Figure 3:**
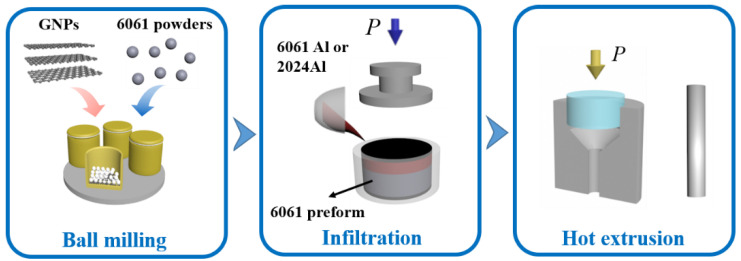
Flow chart of pressure infiltration process for preparing composite materials.

GNPs were firstly mixed with 6061Al powder (the weight ratio of the GNPs and Al powder was 1.0 wt.%) using a planetary mill. Mixed powder after ball milling is shown in Figure 2c. Ball milling of composite powder and the calculation of mass fraction, please refer to the previous article [32]. As shown in Figure 3, the mixture (GNPs/6061Al) powders were then put into a steel mold and further pressed to the set height to prepare the preforms and the volume content of the milled particles in the mold was about 60 vol.%. Preheating temperatures for the preform and pressure infiltration dies were 530 °C and 760 °C, respectively. During the infiltration process, a pressure of 10 MPa was applied and maintained for 8 min, followed by the solidification of the composites in the air, and then the composites were obtained. During the pressure infiltration, we used 2024Al and 6061Al as an infiltration alloy, respectively; therefore, we obtained two different kinds of composites, which were named (GNPs/6061Al)/2024Al and GNPs/6061Al (the specific information is shown in Table 4). Measuring the height of composites, which represent the actual volume contents of the milled particles, accurate amounts of the GNPs contents of the composites have been calculated to be 0.6 wt.%. Afterward, composites samples were extruded at 500 °C with an extrusion ratio of 11:1. Before microstructure observation and properties testing, all samples were annealed at 340 °C for 1 h.

### 2.3. Characterizations and Mechanical Tests 

Morphology of the GNPs and 6061Al powders were characterized by FEI Quanta 200FEG (Thermo Fisher Scientific, Waltham, MA, USA) scanning electron microscope (SEM). Morphology of the (GNPs/6061Al)/2024Al and GNPs/6061Al composites were observed using the Olympus PMG3 (Olympus Corporation, Tokyo, Japan) optical microscope. Raman measurement was characterized by a JY-HR800 (HORIBA Jobin Yvon, Paris, France) laser Raman spectrometer using a 532 nm solid-state laser as an excitation source. X-ray diffraction (XRD) analysis was performed by using a Rigaku D/max-rB diffractometer (Rigaku Corporation, Tokyo, Japan). The specimens were subjected to Cu-Kα radiation (0.15418 nm) with a scanning speed set at 2°/min while the 2θ scans were performed from 20° to 90°. Element analysis of Al, C, Mg, Si, and Cu was identified using the energy dispersive spectrometer (EDS), and the grain size distribution was characterized by electron backscattered diffraction (EBSD) using an FEI Quanta 200FEG SEM. Transmission electron microscope (TEM) observation was performed on Talos f200x (FEI, Hillsboro, OR, USA) transmission electron microscopy. Tensile tests were performed on an Instron 5569 (Instron, Boston, MA, USA) universal electrical tensile testing machine with a cross-head speed of 0.1 mm/min. All the tensile tests have been performed on at least four samples to improve the statistical significance of the results. The fracture surface of the composites was also observed by FEI Quanta 200 SEM.

## 3. Results and Discussion

### 3.1. FEM Simulation and Microstructure Investigation of As-Cast Composites

**Figure 4:**
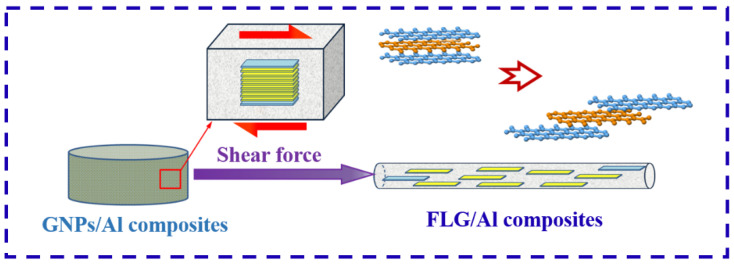
Schematic of the deformation treatment process of the heterogeneous matrix design composite.

The influence of the heterogeneous matrix on the deformation behavior of GNPs is studied by FEM simulation, as shown in Figure 1b. The typical structural unit of heterogeneous matrix GNPs/Al alloy composite was constructed by taking skeleton matrix (A–Al alloy), GNPs (intermediate non-deformable sheet), and continuous matrix (B–Al alloy) as the constituent parts. The strength of continuous B–Al alloy was fixed to 300 MPa. It can be seen that with the decline of the strength of A–Al alloy from 299 MPa to 1 MPa, the shear strain Δε(A-B) between GNPs increases from 0.036 to 0.33, which amounts to nearly 10 times higher. It shows that with the increase in the mechanical properties difference Δσ(A-B) between the skeleton matrix (A–Al alloy) and the continuous matrix (B–Al alloy), obvious asymmetric strain fields are formed on both sides of GNPs. It can significantly promote the dispersion and peeling of GNPs. A schematic of the heterogeneous matrix design composite is shown in Figure 4.

**Figure 5:**
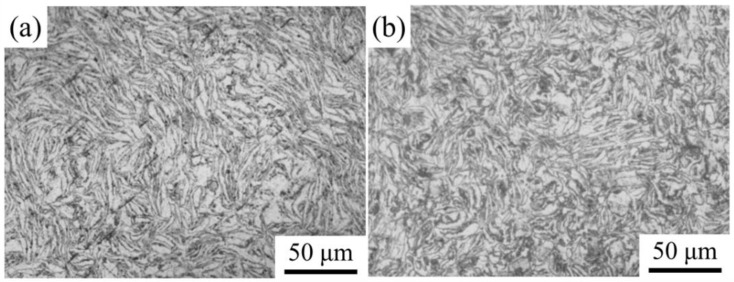
Morphology of the (GNPs/6061Al)/2024Al and GNPs/6061Al composites before the extrusion treatment: (**a**) (GNPs/6061Al)/2024Al; (**b**) GNPs/6061Al.

Morphology of the (GNPs/6061Al)/2024Al and GNPs/6061Al composites before the extrusion treatment have been shown in Figure 5. No significant casting defect was observed, while long strip shape microstructure had been observed in the composites before the extrusion treatment, as shown in Figure 5a ((GNPs/6061Al)/2024Al) and Figure 5b (GNPs/6061Al), respectively. After ball milling, the spherical 6061Al powders were transformed into sheets, which were beneficial for the absorption of the GNPs due to the increase in a specific area [33,34,35]. During the process of consolidation, the shape of 6061Al maintained a sheet shape, and the GNPs distributed along the 6061Al sheets. In addition, no significant difference in the Al grain size has been found between (GNPs/6061Al)/2024Al and GNPs/6061Al composite before the extrusion treatment.

**Figure 6:**
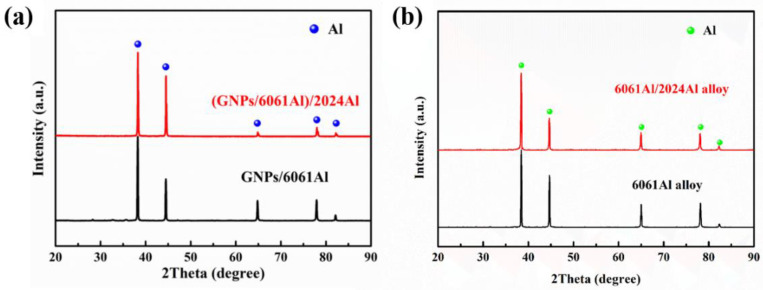
XRD analysis results of the as-cast composites and alloys: (**a**) (GNPs/6061Al)/2024Al and GNPs/6061Al composites; (**b**) 6061Al/2024Al alloys and 6061Al alloys.

XRD analysis results of the as-cast (GNPs/6061Al)/2024Al and GNPs/6061Al composites, as shown in Figure 6. The distinct peaks detected in the composites were at the 2θ angles of 38.47°, 44.72°, 65.10°, 78.23°, and 82.44°, which corresponded to the peaks of Al (38.47°, 44.71°, 65.10°, 78.23°, and 82.44° according to JCPDS #85-1372), respectively. It can be seen that all the peaks correspond to the Al matrix and not any significant peaks from carbides and other phases. This means that the number of carbides in the microstructure is small in both composites. Similar results have been reported in previous work [28]. In addition, one explanation of this phenomenon involves the low extent of carbon material (e.g., graphene, CNTs) used in composites [28].

**Figure 7:**
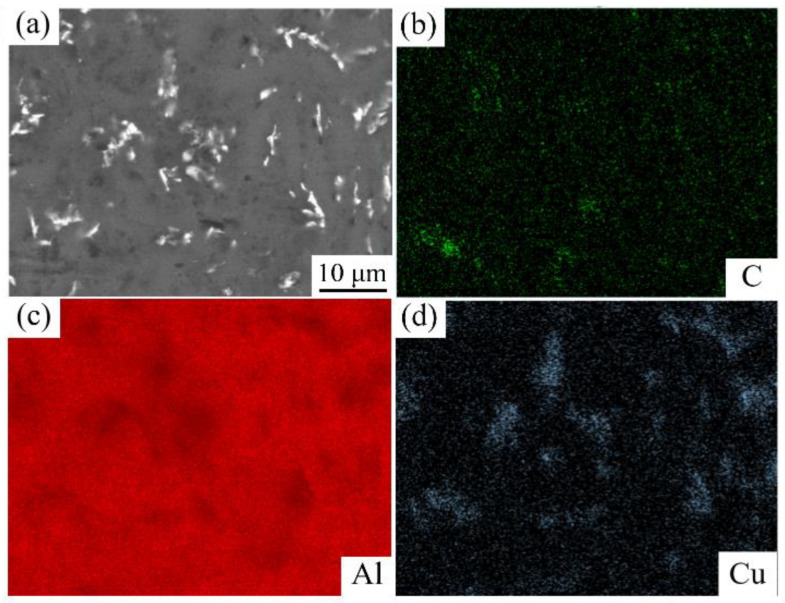
EDS analysis result of as-cast 0.6 wt.% (GNPs/6061Al)/2024Al composite: (**a**) microstructure, (**b**) C element, (**c**) Al element, (**d**) Cu element.

**Figure 8:**
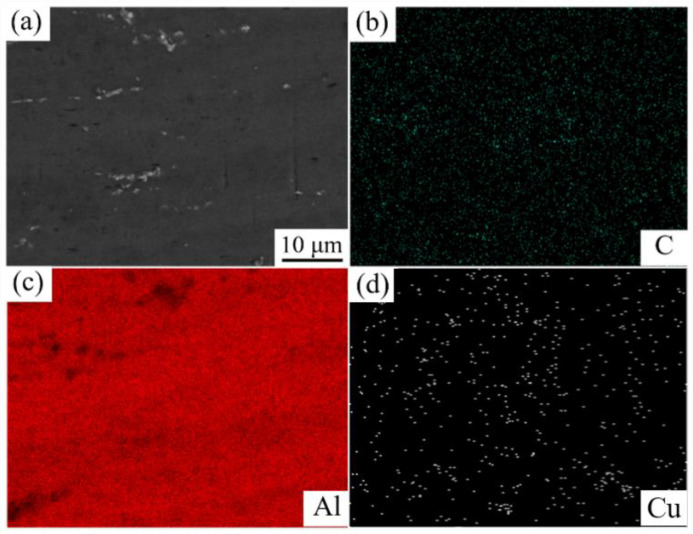
EDS analysis result of 0.6 wt.% (GNPs/6061Al)/2024Al composite after hot extrusion: (**a**) microstructure, (**b**) C element, (**c**) Al element, (**d**) Cu element.

EDS analysis results of the as-cast (GNPs/6061Al)/2024Al are presented in Figure 7. 2024Al belongs to Al–Cu–Mg system aluminum alloy, and 6061Al belongs to Al–Mg–Si. It can be found that the characteristic element in 2024Al is Cu. In the research, we want to distinguish the position of the heterogeneous matrix by comparing characteristic elements. The composition distribution of the composite is obviously uneven. The areas of Cu enrichment in SEM images represent the areas where 2024Al is located due to the fact that the addition of GNPs impedes the diffusion of elements. The mechanical contact between GNPs and aluminum powder is good after ball milling; GNPs are adsorbed and embedded in aluminum powder. After infiltrating into another aluminum matrix (2024Al) by the pressure infiltration method, the element diffusion between the two aluminum alloys is limited due to the isolation effect of GNPs [36]. This is also a key to the realization of the above design idea. The EDS analysis results of the (GNPs/6061Al)/2024Al composite after hot extrusion are presented in Figure 8. The distribution of the C element shows that the dispersion of GNPs is more uniform than that of the as-cast composite after hot extrusion.

### 3.2. Microstructure Evolution of Composites after Deformation Treatment

**Figure 9:**
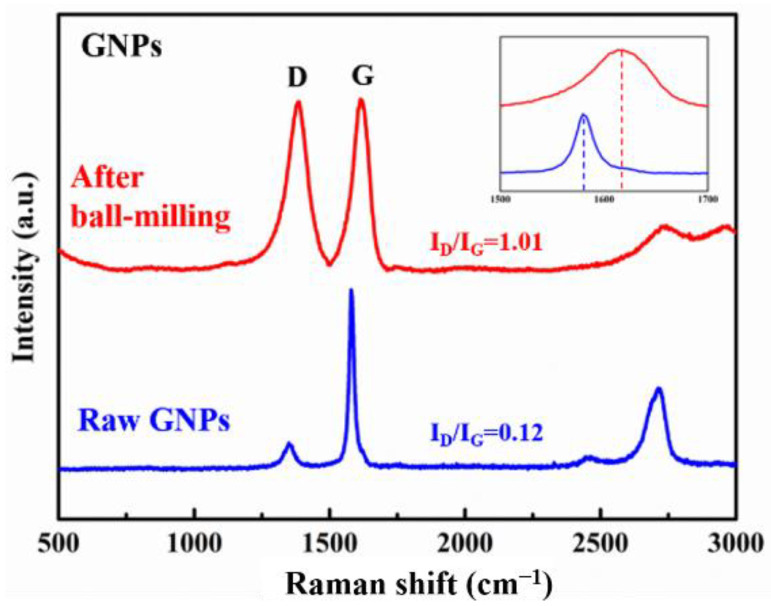
Raman results of raw GNPs and the GNPs after ball milling.

**Figure 10:**
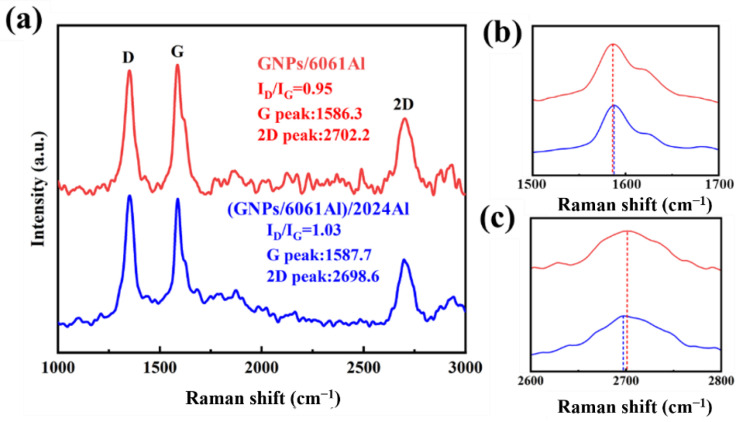
(**a**) Raman results of composites GNPs/6061Al and (GNPs/6061Al)/2024Al composite after extrusion; (**b**,**c**) partial diagram of Raman results.

Typical Raman results of raw GNPs, the GNPs after ball milling are shown in Figure 9; the results of (GNPs/6061Al)/2024Al and GNPs/6061Al composites after hot extrusion are presented in Figure 10. Before characterization, the samples were slightly corroded and cleaned. When the aluminum matrix is characterized by the Raman spectrum, it will produce fluorescence and interfere with the experimental results; therefore, this effect is eliminated in the above way. Raman is an effective analytical method for GNPs; its peak position and intensity reflect the structural information of GNPs. The characteristic peaks of GNPs mainly include the D peak, G peak, and 2D peak. Among them, the D peak relates to the integrity of GNPs, which reflects the defects, and the position of the G peak is closely related to the number of GNPs layers, and the position of the G peak will change with the number of GNPs layers [37]. With the increase in the number of GNPs layers *N*, the position of the G peak will move to the low wavenumber, and its displacement is related to 1/*N*, but the shape of the G peak does not change significantly [10]. Compared with the G peak, it is preferable to use a 2D peak to characterize the number of graphite layers because the shape and position of the 2D peak change with the increase in the number of GNPs layers. With the increase in the number of GNPs layers, the 2D peak becomes wider, the intensity decreases, and there is a trend of redshift. In addition, I_D_/I_G_ is positively correlated with defect density, which can be used to evaluate the defect degree of GNPs; therefore, this study mainly uses I_D_/I_G_ and 2D peak frequency to qualitatively analyze the state of GNPs in composites. As shown in Figure 9, there is a blue shift of the G peak after ball milling. After ball milling, the morphology of GNPs changed significantly compared with before, especially the thickness of GNPs decreased significantly, which is consistent with many reports in the literature [4,7,9,10]. By comparing the Raman spectra of the composites, it can be found that the position of the G peak of heterogeneous composites is higher than that of homogeneous materials, while the position of the 2D peak is lower than that of homogeneous materials. This means that the heterogeneous matrix has a better effect on eliminating GNPs agglomeration and reducing the number of GNPs layers, which is beneficial to the mechanical properties of the composites. In addition, comparing the I_D_/I_G_ of the two composites, it is found that the change of defect degree is not obvious, which may be due to the production of new FLG during hot extrusion. In order to evaluate the effect of the heterogeneous matrix, it is necessary to combine the tests of properties, especially the elastic modulus.

**Figure 11:**
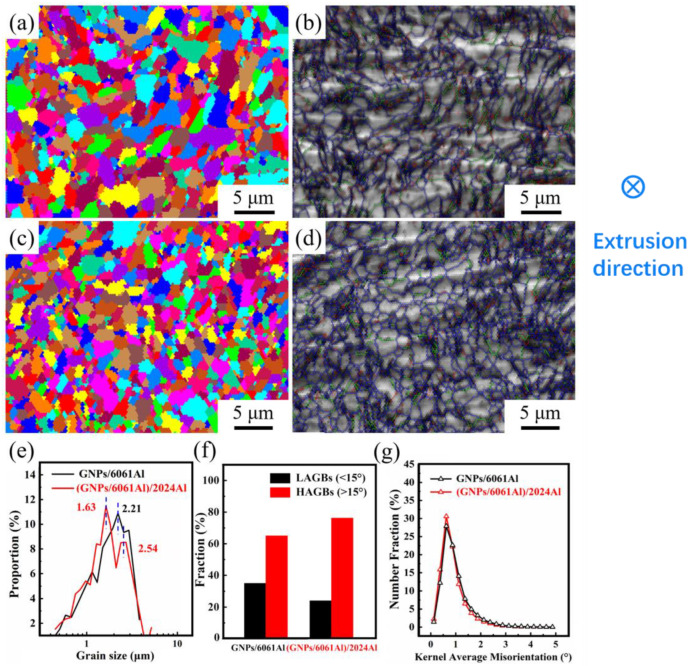
EBSD analysis results of extruded composites: (**a**) the grain map and (**b**) the grain boundary (GB) map of GNPs/6061Al composite; (**c**) the grain map and (**d**) the grain boundary (GB) map of (GNPs/6061Al)/2024Al; (**e**) the distribution of grain size; (**f**) fraction of GB characteristics; (**g**) number fraction of kernel average misorientation (KAM) distribution.

EBSD images of the (GNPs/6061Al)/2024Al and GNPs/6061Al composites after hot extrusion are demonstrated in Figure 11. Hot extrusion is an effective method to optimize the microstructure of materials, which can refine the grains and improve the properties of materials [32]. It is found that the grains of the two hot extruded composites are very fine, almost all the grain sizes are below 5 μm, but the distribution of the grain sizes is different. Furthermore, we can see that the distribution of grain sizes in different matrices has different characteristics: for heterogeneous composites (2024Al matrix, the average grain size is small, and there are fine grains between relatively large grains), while the average grain size of homogeneous composite (6061Al matrix composite) is larger, and there is no fine grain between the grains—shown in Figure 11a,c.

By using OIM analysis software(EBSD-TSL OIM 6.0), the grain sizes of two kinds of materials were counted, and the results are illustrated in Figure 11e. The results of size statistics are in good agreement with the results of grain diagram observation. After hot extrusion, there are two peaks in the grain size distribution curve of heterogeneous composites (this phenomenon also appears in other non-uniform composites), while only one peak appears in the curve of homogeneous composites after hot extrusion. The size of 0.6 wt.% GNPs/6061Al composite is 2.21 μm, in contrast, that of 0.6 wt.% (GNPs/6061Al)/2024Al composite is 1.63 μm and 2.54 μm. This phenomenon is probably due to the existence of two kinds of aluminum alloys in the heterogeneous composites, which results in the difference in grain deformation during the hot extrusion process. The degree of elongation along the extrusion direction and a different degree of reduction in the area perpendicular to the extrusion direction led to the image of large grains mixed with small grains; however, the result shows that the grain size is relatively uniform in the homogeneous composite after deformation, and the fine grains are few.

**Figure 12:**
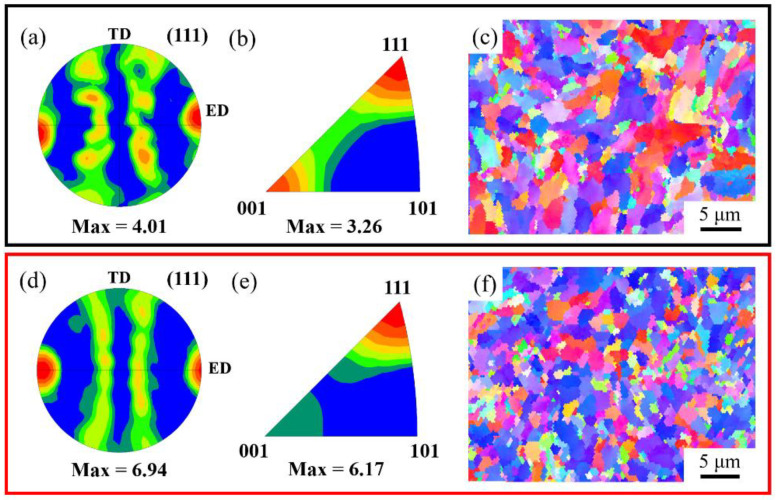
The (111) pole figures of extruded composites (**a**) GNPs/6061Al (**d**) (GNPs/6061Al)/2024Al composite. (**b**) and (**e**) inverse pole figures, and inverse pole figure (IPF) maps of (**c**) GNPs/6061Al (**f**) (GNPs/6061Al)/2024Al.

Figure 11b,d are the grain boundary (GB) maps of two composites. It can be found that there are numerous high-angle grain boundaries (HAGBs, misorientation angle over 15°, marked by dark blue lines) and low-angle grain boundaries (LAGBs, misorientation angle below 15°, green and red lines). The relative fraction of HAGBs and LAGBs is shown in Figure 11f. It can be found that the HAGBs in both composites are more than the LAGBs, and the HAGBs in the heterogeneous composite are more than that in the homogeneous composite. In addition, this phenomenon was reported in the previous literature, which was related to the sub-grains [38]. The results of kernel average misorientation (KAM) distribution are shown in Figure 11g, where the KAM values are narrowly distributed and the mean values are below 1°. The KAM distributions for (GNPs/6061Al)/2024Al and GNPs/6061Al composite are similar, while the distribution of GB misorientations of the heterogeneous composite is even higher than the homogeneous composite. The (111) pole figures and inverse pole figures are shown in Figure 12. The orientation of matrix grains in both extruded composites has a strong <111> fiber texture parallel to ED. While the max strength of the texture in the (GNPs/6061Al)/2024Al composite is higher than the GNPs/6061Al composite.

**Figure 13:**
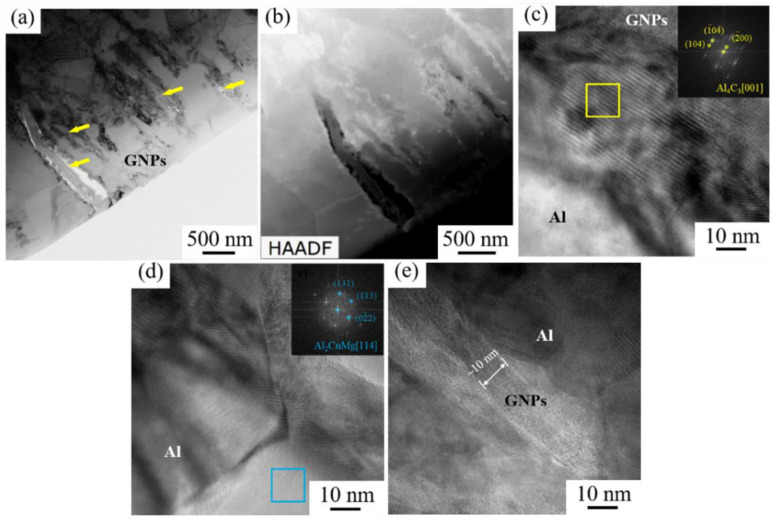
TEM analysis results of (GNPs/6061Al)/2024Al composite after extrusion: (**a**) Bright-field image; (**b**) HADDF image; (**c**–**e**) high magnification images, the inset of (**c**,**d**) FFT—the result of the selected area.

TEM microstructures of the (GNPs/6061Al)/2024Al composite have been shown in Figure 13. It is found from Figure 13a that the GNPs are mainly distributed at the grain boundary of the aluminum alloy matrix, and there is a debonding of the interface, which may be caused by excessive brittle phases generated by reaction; the Al_4_C_3_ can be observed in Figure 13c. In comparison, it can be observed from Figure 13d that after solution aging, the Al_2_CuMg phase appears near the Al grain in the composite. It is a type of nano-scaled precipitation phase in 2024Al, which is conducive to the strengthening of the material; however, it is worth mentioning that most of the interfaces are well bonded, as shown in Figure 13e. It can be seen that this piece of GNP is thinner than the raw material, which can play a better effect in transmitting load, which is beneficial to obtaining composites with high mechanical properties.

### 3.3. Mechanical Properties of Composites

**Figure 14:**
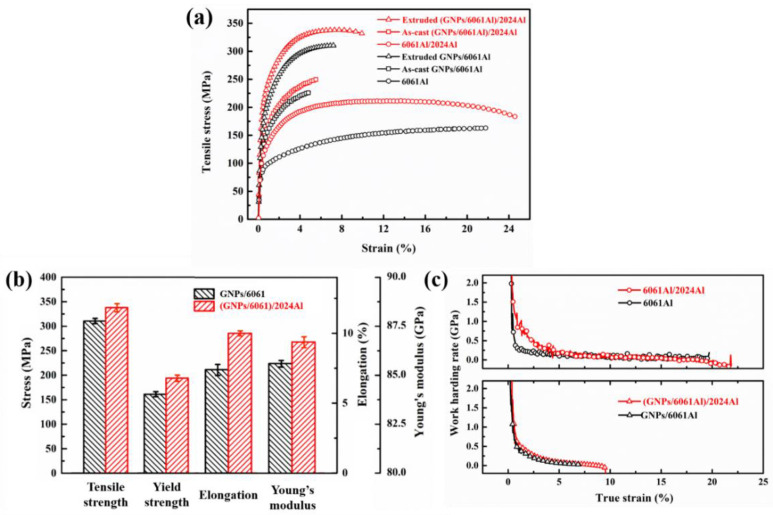
Representative mechanical properties of the composites and matrixes after extrusion: (**a**) tensile stress–strain curves; (**b**) comparison of the tensile strength, yield strength, elongation, and Young’s modulus; (**c**) strain hardening curves calculated from stress–strain curves.

Representative tensile curves of the (GNPs/6061Al)/2024Al and GNPs/6061Al composites, together with the matrix alloys, are shown in Figure 14a. The mechanical properties of the composite are presented in Figure 14b and Table 5. The tensile strength of 0.6 wt.% (GNPs/6061Al)/2024Al composite is 338.1 MPa, while that of 0.6% GNPs/6061Al composite is 310.5 MPa. By comparison, it is found that the heterogeneous composite ((GNPs/6061Al)/2024Al) has certain advantages, which are related to the precipitation phase introduced by 2024Al in the infiltration process. As shown in Figure 11b,d, there are more HAGBs in the (GNPs/6061Al)/2024Al composites, which will effectively hinder the movement of dislocations, the movement across grains. Furthermore, as mentioned in Section 1 [10], the peeling of the GNPs can enhance the specific surface area of the reinforcement, which means that the reinforcing effect improves and the strength of composites increases. After extrusion treatment, the mechanical properties of the two composites were improved. Hot extrusion optimizes the microstructure of the material, refines the internal grains of the material, eliminates the agglomeration of graphene, and increases the plasticity and elastic modulus of the material.

Moreover, it should be noted that (GNPs/6061Al)/2024Al composite also has obvious advantages in elongation and elastic modulus, especially for the change of elastic modulus. Deformation treatment cannot bring a significant change of elastic modulus for dense aluminum alloy matrix. The elastic modulus of the heterogeneous composite ((GNPs/6061Al)/2024Al) is 86.7 GPa, which is slightly higher than that of the homogeneous composite GNPs/6061Al. The elastic modulus is mainly related to the density and the intrinsic properties of the material. Through the same deformation treatment, the composites have reached a high relative density. So, in this case, the elastic modulus is mainly affected by the intrinsic properties of the material. According to the literature [39,40], the deformation treatment can eliminate the aggregation of GNPs in the composite and even peel the GNPs sheet layer (when the raw material is a multi-layer GNPs). Based on the analysis above, it is considered that the difference in elastic modulus of the two composites after deformation treatment is mainly caused by the additional non-uniform deformation caused by the heterogeneous matrix. In addition, there is not any obvious difference in the work hardening behavior between the two composites, while the 6061Al/2024Al heterogeneous matrix demonstrates higher strain hardening behavior than the 6061Al matrix. Furthermore, the heterogeneous matrix design strategy not only obtains the mixed structure of coarse and fine grains together with greater texture strength shown in Figure 12a,d, but also introduces an additional hardening mechanism, thus increasing the tensile plasticity. This is considered to be the reason why the plasticity of (GNPs/6061Al)/2024Al is better than GNPs/6061Al, which is the homogeneous matrix. There are few reports on the preparation of heterogeneous matrix composites by pressure infiltration technology, and the deformation mechanism still needs to be further studied in the future. In order to improve the intrinsic trade-off of strength and toughness of metal materials, mainstream research is also focused on microstructure design [41,42,43].

**Figure 15:**
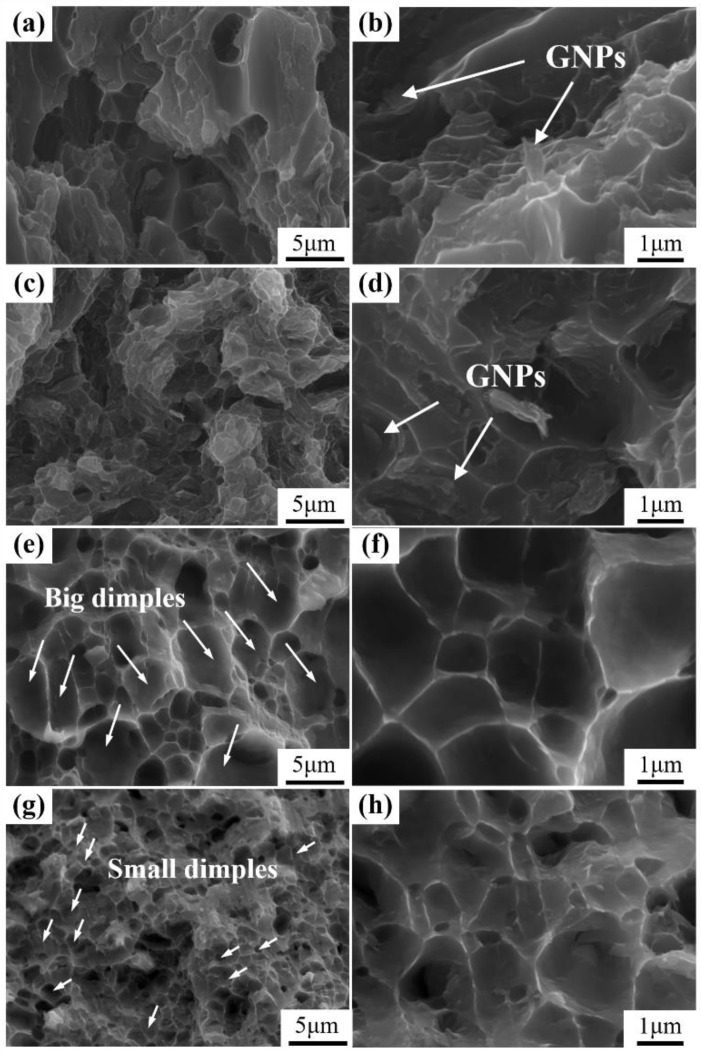
Fracture surfaces of the extruded composites: (**a**,**b**) GNPs/6061Al composite; (**c**,**d**) (GNPs/6061Al)/2024Al composite; (**e**,**f**) 6061Al matrix alloy; (**g**,**h**) 6061Al/2024Al matrix alloy.

The fracture surfaces of the (GNPs/6061Al)/2024Al and GNPs/6061Al composites after hot extrusion have been shown in Figure 15a–d. It is found that the fracture characteristics of composites are mainly dimples with a number of tear ridges. The shape and trend of dimples are irregular, which indicates the superior plasticity of the composites; however, the dimples are due to the distribution of GNPs, and the crack propagation around the GNPs forms dimples of different sizes and shapes. In addition, it can be viewed on the magnification images, shown in Figure 15b,d, that some GNPs were pulled out (marked by the arrow in the figure), which serves to transfer the load. In addition, this phenomenon is supposed to impede the propagation of cracks. In contrast, the fracture surfaces of the matrix alloys were observed, as shown in Figure 15e–h. There are a large number of small dimples in the fracture surface of heterogeneous matrix 6061Al alloy, while the dimples are larger in homogeneous matrix 6061Al/2024Al alloy, which is consistent with the results in composites.

### 3.4. Strengthening Mechanism of GNPs/Al Composites

The possible strengthening mechanisms of particle-reinforced composites have been discussed in previous studies [44]. Load transfer from Al matrix to reinforcements (∆*σ_L_*); grain refinement results from the pinning effect of GNPs (∆*σ_G_*_(*R*)_); the thermal mismatch mechanism is caused by the generation of dislocations due to the different coefficient of thermal expansion (CTE) between Al matrix and the reinforcements (∆*σ_T_*); the Orowan strengthening mechanism is related to the Orowan looping system (∆*σ_Oro_*); therefore, the multiple strengthening mechanisms operating in the composite can be expressed as Equation (2). The following calculation will take (GNPs/6061Al)/2024Al composite as the research object.

(2) $$\sigma_{c}=\sigma_{m}+\Delta \sigma_{G\left(R\right)}+\Delta \sigma_{T}+\Delta \sigma_{Oro}+\Delta \sigma_{L}$$

The strengthening effects of grain refinement can be calculated by the Hall–Petch formula, as Equation (3):

(3) $$\Delta \sigma_{G\left(R\right)}=K\left(d_{1}{}^{-\frac{1}{2}}-d_{0}{}^{-\frac{1}{2}}\right)$$

where K is a constant for Al, which is 0.04 MPa·m^1/2^ [45]; *d* is the average grain size of the composite after hot extrusion, as shown in Figure 11, and it can be measured as 2.01 μm. So, the results show that the contribution of the grain refinement effect is about 9.1 MPa. This effect is mainly related to the inhibition of reinforcement on grain grown during the preparation process and the hot extrusion.

While there are CTE differences between reinforcement and matrix, a number of dislocations may generate on the interface during the cooling process at the end of hot extrusion. The new dislocations make a significant contribution to the composites. The CTE of GNPs is −8 × 10^−6^/K and 23.6 × 10^−6^/K for the matrix Al. The contribution of the thermal mismatch mechanism to tensile strength can be estimated by the following formula [46]:

(4) $$\Delta \sigma_{CTE}=kG_{m}b\sqrt{12\frac{\Delta \alpha \Delta TV_{G}}{bd_{G}}}$$

where k is a constant (0.5) and b is the Burgers vector of matrix Al (0.286 nm). *G_m_* is the shear modulus of the matrix and can be calculated using the basic parameters of Al. According to the literature, the *G_m_* is about 27.5 GPa [46]; ∆*α* is the difference in CTE between the two parts, matrix, and reinforcement; ∆*T* is the gradient in the temperature from hot extrusion (500 °C) to the ambient temperature (25 °C). In addition, the *V_R_* and *d_R_* are the volume fraction and approximate diameter of reinforcements, respectively; *d_R_* can be obtained from microstructure observation. According to Equation (4), the contribution of the thermal mismatch mechanism is about 9.7 MPa.

The particles in the composites can inhibit the movement of dislocation and this may enhance the strength of materials. This mechanism plays an important part in the strengthening of material, which can be described by the Orowan formula (Orowan–Ashby equation [47]), as Equation (5):

(5) ΔOro=0.13GmbdR12VG13−1lndR2b

where *G_m_* and b are the shear modulus and the Burgers vector of matrix Al. *V_R_* is the volume fraction of reinforcements in the composite. The result indicates that the contribution from the Orowan looping system is about 24.2 MPa.

Through calculation, it can be concluded that the strengthening effect of load transfer is about 47.0 MPa. From the above results, it can be seen that load transfer strengthening is the most important contribution. Compared with other strengthening mechanisms, it is related to the addition of GNPs.

## 4. Conclusions

In the present work, the stress field of the heterogeneous matrix under the condition of thermal deformation was researched by using FEM. In addition, heterogeneous 0.6 wt.% (GNPs/6061Al)/2024Al and homogeneous 0.6 wt.% GNPs/6061Al composites were prepared and hot extruded due to the advantages of pressure infiltration technology. In the process of deformation treatment, due to the difference in properties of the two alloys, an extra non-uniform stress field is produced, which eliminates the aggregation of GNPs in the process. Raman test was performed on the composites before and after extrusion. It finds that the peak position of the D peak uplifted after extrusion. The interlayer slide of GNPs was observed by TEM. The tensile strength of extruded (GNPs/6061Al)/2024Al composite is 338.1 MPa, which is higher than 310.5 MPa of GNPs/6061Al composite. Moreover, the composites have obvious advantages in elongation and elastic modulus, especially the change of the elastic modulus. The elastic modulus of heterogeneous composite (GNPs/6061Al)/2024Al is 86.7 GPa, which is slightly higher than that of homogeneous composite. Excluding the factors that affect the elastic modulus, such as relative density, it is considered that the heterogeneous matrix brings an extra non-uniform stress field during the deformation process. This improves the dispersion of GNPs and produces the few-layer graphene, thus enhancing the strengthening effect of GNPs and improving the performance of the composite. Moreover, the heterogeneous matrix design strategy not only obtains the mixed structure of coarse and fine grains together with greater texture strength but also introduces an additional hardening mechanism, thus increasing the tensile plasticity and improving the intrinsic trade-off of strength and toughness.

## Figures and Tables

**Table 1:** The work hardening curve of aluminum alloys in FEM.

Stress (MPa)	$\sigma_{y}$	$\sigma_{y}+50$	$\sigma_{y}+100$	$\sigma_{y}+120$
Plastic strain	0	0.5	1.5	2

**Table 2:** The mechanical properties of GNPs and Al alloy in FEM.

	Young’s Modulus	Poisson’s Ratio	Density
GNPs	1000 GPa	0.1	2.25 g/cm^3^
Al alloy	70 GPa	0.3	2.7 g/cm^3^

**Table 3:** Chemical compositions of 6061Al powder and 2024Al (wt.%).

**Element**	**Mg**	**Si**	**Cu**	**Fe**	**Zn**	**Al**
6061Al powder	1.12	0.75	0.32	0.65	0.22	Bal.
**Element**	**Cu**	**Mg**	**Mn**	**Zn**	**Cr**	**Al**
2024Al	4.05	1.65	0.75	0.22	0.07	Bal.

**Table 4:** Specific information of (GNPs/6061Al)/2024Al and GNPs/6061Al preparation.

Process	Ball Milling	Pressure Infiltration
(GNPs/6061Al)/2024Al	6061Al powder + GNPs	2024Al
GNPs/6061Al	6061Al powder + GNPs	6061Al

**Table 5:** Tensile properties of composites and matrices after hot extrusion and annealing treatment.

Specimen	Condition	YS (MPa)	UTS (MPa)	EI. (%)	E (GPa)
0.6 wt.%(GNPs/6061Al)/2024Al	Extruded	194.2 ± 7.3	338.1 ± 9.2	10.0 ± 1.1	87.2 ± 0.2
0.6 wt.%GNPs/6061Al	Extruded	161.1 ± 5.2	310.5 ± 4.5	7.4 ± 1.4	86.7 ± 0.3
0.6 wt.%(GNPs/6061Al)/2024Al	As-cast	125.8 ± 2.6	249.5 ± 6.1	5.7 ± 0.8	83.2 ± 0.2
0.6 wt.%GNPs/6061Al	As-cast	119.3 ± 3.8	227.3 ± 3.2	5.0 ± 1.1	82.8 ± 0.1
6061Al/2024Al	Extruded	102.8 ± 6.1	207.4 ± 5.7	24.8 ± 1.6	82.5 ± 0.3
6061Al alloy	Extruded	71.3 ± 5.8	161.5 ± 7.1	21.9 ± 2.7	79.6 ± 0.1

## Data Availability

Not applicable.
